# Fast and Accurate Finite Transducer Analysis Method for Wireless Passive Impedance-Loaded SAW Sensors

**DOI:** 10.3390/s18113988

**Published:** 2018-11-16

**Authors:** Wei Luo, Yang Yuan, Yi Wang, Qiuyun Fu, Hui Xia, Honglang Li

**Affiliations:** 1 Engineering Research Center for Functional Ceramics of the Ministry of Education, School of Optical and Electronic Information, Huazhong University of Science and Technology, Wuhan 430074, China; hustluowei@gmail.com (W.L.); yuanyang0125@126.com (Y.Y.); ghost-zzz@163.com (Y.W.); fuqy@mail.hust.edu.cn (Q.F.); 2 Institute of Electrical Engineering, Chinese Academy of Sciences, No. 6 Beiertiao, Zhongguancun, Beijing 100190, China; 3 Institute of Acoustics, Chinese Academy of Sciences, 21 North 4th Ring Road, Haidian District, Beijing 100190, China

**Keywords:** surface acoustic wave (SAW) sensor, wireless passive impedance-loaded SAW sensor, boundary element method, finite element method

## Abstract

An accurate and fast simulation tool plays an important role in the design of wireless passive impedance-loaded surface acoustic wave (SAW) sensors which have received much attention recently. This paper presents a finite transducer analysis method for wireless passive impedance-loaded SAW sensors. The finite transducer analysis method uses a numerically combined finite element method-boundary element method (FEM/BEM) model to analyze non-periodic transducers. In non-periodic transducers, FEM/BEM was the most accurate analysis method until now, however this method consumes central processing unit (CPU) time. This paper presents a faster algorithm to calculate the bulk wave part of the equation coefficient which usually requires a long time. A complete non-periodic FEM/BEM model of the impedance sensors was constructed. Modifications were made to the final equations in the FEM/BEM model to adjust for the impedance variation of the sensors. Compared with the conventional method, the proposed method reduces the computation time efficiently while maintaining the same high degree of accuracy. Simulations and their comparisons with experimental results for test devices are shown to prove the effectiveness of the analysis method.

## 1. Introduction

Wireless passive surface acoustic wave sensors have been investigated since the 1990s because they have several advantages, such as, no need for power supply, good environmental durability, high sensitivity, etc. [1]. There are various types of surface acoustic wave (SAW) sensors, pressure sensors [2], gas sensors [3], torque sensors [4], etc. Compared with them, impedance-loaded surface acoustic wave sensors offer a much wider application range [5,6]. These sensors use the SAW chip as a transponder and the load sensing part can be any impedance-varying sensor that matches the scope of impedance variation. Impedance-loaded sensors with a wide application range have received much attention in recent years [7,8,9].

**Figure 1:**
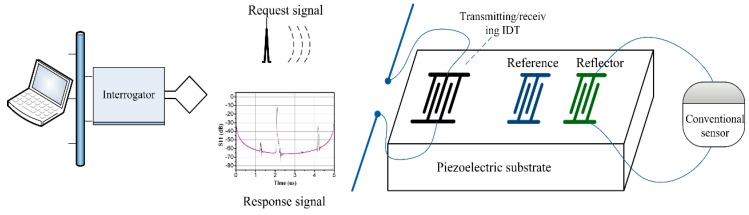
Schematic of an impedance-loaded surface acoustic wave (SAW) sensor.

Conventional impedance-loaded sensors have two types of structures: The delay line structure and resonator structure [6,7,10,11]. The basic construction of delay line structure sensors is shown in Figure 1. The SAW transponder is composed of several interdigital transducers (IDTs), one of which is used as a transmitting/receiving IDT responsible for transmitting and receiving wireless signals and the others are used as reflectors. One of the reflectors is loaded with conventional sensors whose varying impedance can be of any type, resistance, capacitance, or inductance. The wireless interrogating electromagnetic wave signal is received by the antenna and transformed to a surface acoustic wave by the transmitting/receiving IDT. The surface acoustic wave propagates along the substrate surface and is reflected by the reflectors. At the same time, the reflection coefficient would be influenced by the externally loaded sensors impedance. The returned surface acoustic wave is transformed back into an electromagnetic (EM) wave by the transmitting/receiving IDT and sent by the antenna. By changing the impedance of outside conventional sensors (electrical boundary condition), the scattering parameters S_11_ of the sensors also changed, which can be detected by the interrogators.

Impedance-loaded SAW sensors operate at high frequency and should be designed to maintain low loss in order to achieve wireless measurement. Therefore, the sensor design must be based on an accurate model. The numerical combined FEM/BEM model is now widely used to derive the influence of device parameters on the frequency response in the design of SAW devices [12,13]. It is more accurate than other conventional models like the equivalent circuit model (ECM) model, this is because it includes the second order effects which other models do not consider. The FEM/BEM model is comprised of a periodic model and a non-periodic model.

The periodic FEM/BEM model can be used to calculate the characteristics of periodic structures, from which more accurate and flexible coupling-of-modes (COM) parameters can be derived. In our previous work [14,15], the COM model of impedance-loaded SAW sensors was constructed based on the models of SAW IDT. The COM parameters, whose accuracy determines the accuracy of the COM model, were calculated using a periodic FEM/BEM model. Next, the SAW device admittance can be derived by the COM model. Moreover, the effects of the impedance matching circuit and the loaded conventional sensors are included in the model.

The non-periodic FEM/BEM model is derived from the basic laws of physics such as Newton’s second law, piezoelectric constitutive equations and Maxwell equations to form a set of system equations. The admittance of a SAW device can be directly derived from the solutions of the system equations with its geometrical characteristics, materials properties, and mechanical and electrical boundary conditions known. The non-periodic FEM/BEM model is more accurate than the COM model for radio frequency (RF) devices because it includes most of the second order effects, but it requires more computing time. In this model, calculating the coefficients of the system equations, which involves double numerical integrals and solving the equations, takes most of the central processing unit (CPU) time. The main calculation content can be divided into four parts, among which the bulk wave contribution takes a long computation time, for it requires the calculation of the general Green’s function at all frequencies. A huge amount of computing time will be needed if the adaptive quadrature method was adopted. Weibiao Wang et al. presented a method based on the sinusoidal weighted integral [16]. However, the case when the integral variable tends to zero was not mentioned. In our paper, this case was carefully considered and the whole numerical properties in the local area were treated accordingly. Our new algorithm leads to a half reduction in required computation time.

When the non-periodic FEM/BEM model is applied to impedance-loaded SAW sensors, the calculation is more time consuming. The loaded impedance is varying, which changes the electrical boundary condition of the IDT. This means that every time the loaded impedance is changed the model calculation needs to be performed. When the electrical boundary condition changes, the complete system equation should be solved once, which requires a long time for solving the large dimension linear equations. For the sensor performance calculation, the loaded impedance might be assigned values twenty times, which leads to the system equation requiring to be solved twenty times. Thus, the calculated cost will be considerably increased. In this paper, a finite transducer analysis method using a non-periodic FEM/BEM model was introduced to simulate impedance-loaded SAW sensors. A fast algorithm was introduced to calculate the coefficients of the system equations. It was shown that, after some modification to the non-periodic model, the sensor admittance can be obtained at any external loaded impedance by using the network theory after performing the calculation for only two states: Short circuit and open circuit. Section 2 provides a short introduction to the non-periodic FEM/BEM model. Section 3 describes in detail an accurate and fast algorithm to calculate the bulk wave part of the equation coefficient. Section 4 and Section 5 present the method that is used to calculate the sensor response at any outside sensor impedance. Section 6 illustrates the simulated results of a test device with different structures. Comparison of the simulated results to the measured results showed good accordance and Section 7 presents the conclusion that our finite transducer analysis of the impedance-loaded SAW sensors is valid.

## 2. Basics of the Non-Periodic FEM/BEM Model

**Figure 2:**
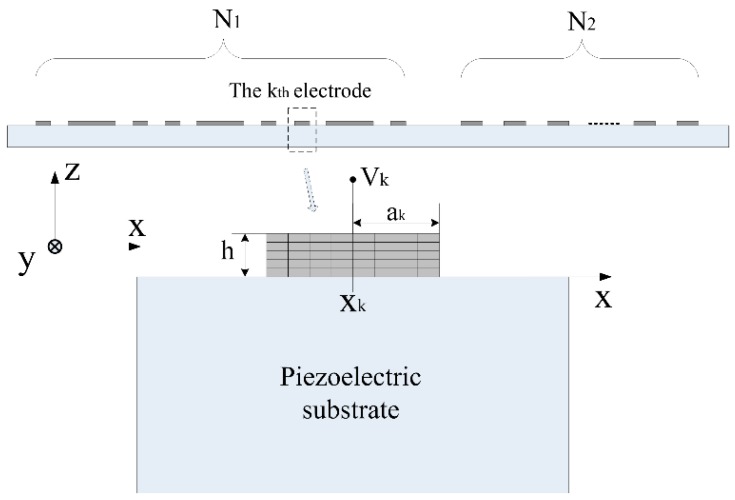
Schematic drawing of the electrodes array.

As shown in Figure 2, the structure is composed of a semi-infinite piezoelectric substrate extending in the half space *z* < 0, whose surface is loaded with metal strip gratings. The grating electrodes are parallel to the y axis and are assumed to be long enough along the *y* axis such that any dependence on *y* can be ignored. Throughout this paper, the time harmonic factor exp (*jωt*) will be omitted.

It is supposed that there are *N_e_* electrodes in the array. The first IDT has *N*_1_ electrodes and the second IDT has *N*_2_ electrodes. Obviously, *N_e_* equals the sum of *N*_1_ and *N*_2_. The location of the *k*th electrode is *X_k_*, the width of the *k*th electrode is 2*a_k_*, and the applied potential on the *k*th electrode is *V_k_*. Because stress and charge distributions are zero outside the electrode-substrate interface, the three components (mechanical displacement, u; stress vector, t*_s_*; electrical potential, ϕ) and the surface charge density σ can be related using the general Green’s function *G(x)* [17]:

(1) $$\left(\begin{array}{c} u(x)\\ \phi (x) \end{array}\right)=\int_{-\infty }^{+\infty }G(x-x\prime )(\begin{array}{c} t_{s}(x\prime )\\ \sigma (x\prime ) \end{array})dx\prime ,$$

The distributions of stress and free charge are expanded using the first-kind Chebyshev polynomial *T_n_*(*x*) for the *k*th electrode:

(2) $$\left(\begin{array}{c} t_{s}(x)\\ \sigma (x) \end{array}\right)=\sum_{n=0}^{N_{k}^{ch}}\left(\begin{array}{c} B_{kn}^{t}\\ B_{kn}^{\sigma } \end{array}\right)\frac{T_{n}(x_{k})}{\sqrt{1-x_{k}^{2}}},\;x_{k}=\frac{x-X_{k}}{a_{k}},\;\begin{array}{l} k=1,\ldots ,N_{e}\\ n=0,\ldots ,N_{k}^{ch} \end{array},$$

where $N_{k}^{ch}$ is the number of Chebyshev polynomials used to expand stress and charge on the *k*th electrode. By incorporating Equation (2) in Equation (1) and timing the Chebyshev polynomials $\frac{T_{i}(x_{j})}{\sqrt{1-x_{j}^{2}}}$ at both sides of Equation (1), we obtain

(3) $${\left(\begin{array}{c} c_{j}^{u}\\ c_{j}^{\phi } \end{array}\right)}_{i}=\sum_{k=1}^{N_{e}}\sum_{n=0}^{N_{k}^{ch}}Y_{kn}^{ij}\left(\begin{array}{c} B_{kn}^{t}\\ B_{kn}^{\sigma } \end{array}\right)\;\begin{array}{c} j,k=1,\ldots ,N_{e}\\ i,n=0,\ldots ,N_{k}^{ch} \end{array},$$

where

(4) $$Y_{kn}^{ij}=a_{k}\int_{-1}^{1}\int_{-1}^{1}G(a_{j}x_{j}-a_{k}x_{k}+\Delta_{jk})\frac{T_{i}(x_{j})}{\sqrt{1-x_{j}^{2}}}\frac{T_{n}(x_{k})}{\sqrt{1-{x_{k}}^{2}}}dx_{j}dx_{k},\;\Delta_{jk}=X_{j}-X_{k},$$

(5) $${\left(\begin{array}{c} c_{j}^{u}\\ c_{j}^{\phi } \end{array}\right)}_{i}=\int_{-1}^{1}\left(\begin{array}{c} u(a_{j}x_{j}+X_{j})\\ \phi (a_{j}x_{j}+X_{j}) \end{array}\right)\frac{T_{i}(x_{j})}{\sqrt{1-{x_{j}}^{2}}}dx_{j},$$

The key point in the numerical calculation of the non-periodic FEM/BEM model is calculating the equation coefficient $Y_{kn}^{ij}$ in Equation (3). Because *G(x)*, which is Green’s function in the space domain, is numerically calculated, its double integral should also be numerically calculated. It is necessary to decompose the function into a sum of various contributions (electrostatic contribution, $G^{0}(x)$; surface wave contribution, $G^{s}(x)$; asymptotic contribution, $G^{\infty }(x)$; bulk wave contribution, $G^{b}(x)$) due to its extremely fast variations [16].

(6) $$G(x)=G^{0}(x)+G^{s}(x)+G^{\infty }(x)+G^{b}(x),$$

Incorporating Equation (6) in Equation (4) leads to

(7) $$Y_{kn}^{ij}={\left(Y_{kn}^{ij}\right)}^{0}+{\left(Y_{kn}^{ij}\right)}^{s}+{\left(Y_{kn}^{ij}\right)}^{\infty }+{\left(Y_{kn}^{ij}\right)}^{b},$$

where ${\left(Y_{kn}^{ij}\right)}^{0}$, ${\left(Y_{kn}^{ij}\right)}^{s}$, ${\left(Y_{kn}^{ij}\right)}^{\infty }$ and ${\left(Y_{kn}^{ij}\right)}^{b}$ are the equation coefficients related to electrostatic contribution, surface wave contribution, asymptotic contribution and bulk wave contribution, respectively. All the above parts can be easily integrated by numerical methods [12,13,16] except the bulk wave contribution. The bulk wave contribution relates to the computation of the general Green’s function at all frequencies, of which the integrated expression requires a long computing time. In the next section, an accurate and fast algorithm for computing the bulk wave contribution part of $Y_{kn}^{ij}$ is presented.

## 3. Accurate and Fast Algorithm for Computing the Bulk Wave Contribution Part of $Y_{kn}^{ij}$

The bulk wave contribution ${\left(Y_{kn}^{ij}\right)}^{b}$ can be expressed as [16]:

(8) $${\left(Y_{kn}^{ij}\right)}^{b}=j^{n-k}\pi a_{j}\int_{-s_{m}}^{s_{m}}\frac{{\bar{H}}^{b}(s)}{\left|s\right|}\exp(-js\omega \Delta )J_{k}(s\omega a_{i})J_{n}(s\omega a_{j})ds,$$

where $\Delta =X_{i}-X_{j}$ is the distance between the *i*th and the *j*th electrodes. *s* is the slowness, and ω is the angular frequency. In Equation (8), the calculation of $\frac{{\bar{H}}^{b}(s)}{\left|s\right|}$, which is the bulk wave part of the Green’s function, requires most of the computing time. The property that the Green’s function is only dependent on the slowness for homogeneous substrates makes it possible that the computation of $\frac{{\bar{H}}^{b}(s)}{\left|s\right|}$ can be done only once. Then the Green’s function can be obtained for all cases on the same substrate simply by interpolation using Chebyshev expansion.

**Figure 3:**
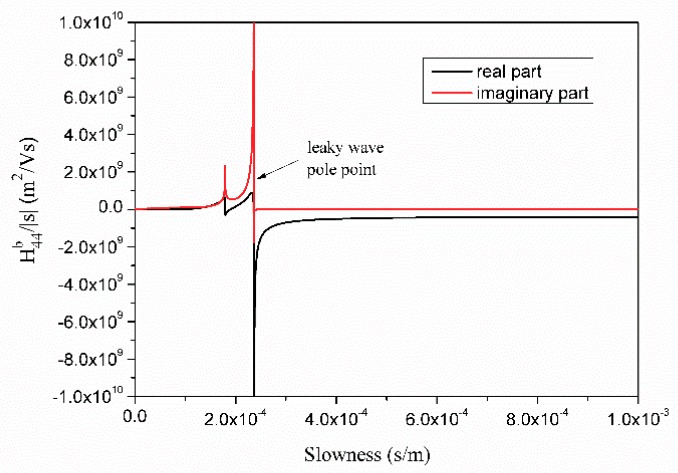
Plot of function $\frac{{\bar{H}}_{44}^{b}(s)}{\left|s\right|}$ when s ∈ [0, 1 × 10^−3^].

**Figure 4:**
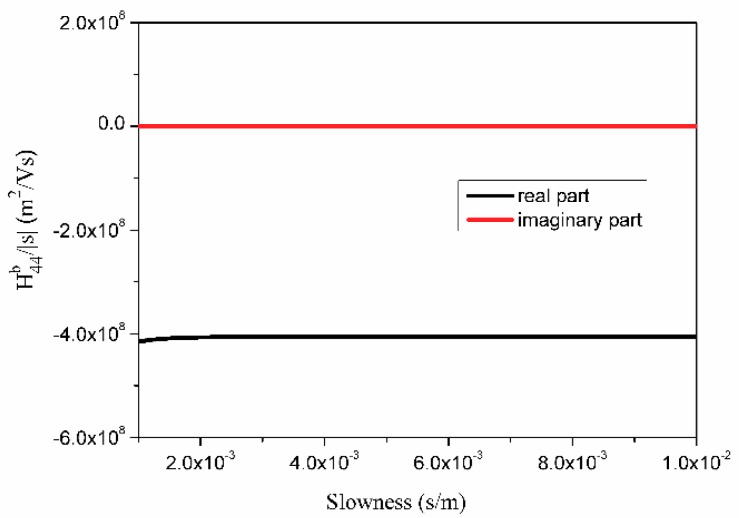
Plot of function $\frac{{\bar{H}}_{44}^{b}(s)}{\left|s\right|}$ when s ∈ [1 × 10^−3^, 1 × 10^−2^].

The values of $\frac{{\bar{H}}^{b}(s)}{\left|s\right|}$ in the slowness domain vary within wide limits. For example, Figure 3 shows the variation of $\frac{{\bar{H}}_{44}^{b}(s)}{\left|s\right|}$ in the scope of $s\in (0,1\times 10^{-3})$ for 42° LiTaO_3_ on which the leaky wave exists. There are still some sharp changes and turns near the leaky wave pole point and branch points after removing the surface wave part of the Green’s function. The function must interpolate in several intervals in the slowness domain to ensure accuracy and lower interpolation expansion numbers. The larger the amplitude of function variation, the greater the interval number. As shown in Figure 4, $\frac{{\bar{H}}_{44}^{b}(s)}{\left|s\right|}$ is monotonic and asymptotic when $s>1\times 10^{-3}$. So, fewer sections can be divided for interpolation in that area. At each interval, $\frac{{\bar{H}}^{b}(s)}{\left|s\right|}$ is monotonic and smooth. The interval number is 2*N* + 1, denoted as −*s_m_* = *s* − *N* < *s* − *N* + 1 <, …, <*s*_0_ = 0 < *s*_1_, …, <*s_N_* = *s_m_*.

The Chebyshev expansion of $\frac{{\bar{H}}^{b}(s)}{\left|s\right|}$ is performed in the *Q*th interval [*s_Q_*, *s_Q+_*_1_],

(9) $$\frac{{\bar{H}}^{b}(s)}{\left|s\right|}=\sum_{p=0}^{N_{Q}}{\bar{c}}_{p}^{Q}{\left(\frac{s-s_{oQ}}{\Delta_{Q}}\right)}^{p},$$

${\bar{c}}_{p}^{Q}$ are the expansion coefficients of Chebyshev polynomials and they only need to be calculated once. The expansion numbers $N_{Q}$ can be 8–10 for large intervals and 3–5 for small intervals.

Substituting Equation (9) into Equation (8), we can get

(10) $${\left(Y_{kn}^{ij}\right)}^{b}=\sum_{p=0}^{N_{Q}}{\bar{c}}_{p}^{Q}\int_{s_{Q}}^{s_{Q+1}}{\left(\frac{s-s_{oQ}}{\Delta_{Q}}\right)}^{p}J_{k}(s\omega a_{i})J_{n}(s\omega a_{j})\exp(-js\omega \Delta )ds,$$

Equation (10) can be decomposed into typical sine or cosine weighted integration forms. Then, the adaptive quadrature method could be applied to approximate the integration. 

In the general case when the electrode number is greater than 100, sωΔ could be larger than 200 for long transducer RF SAW devices. The integral of Equation (10) is an oscillatory integral, where the computation cost is proportional to the value of sωΔ. When the scale of sωΔ is larger than 1 × 10^4^, the accuracy of the adaptive quadrature is poor. 

A robust and accurate algorithm is needed to calculate the appropriate cases for all values of sωΔ. The best way to solve this problem is by expanding the integrand to an analytic series. Thus, another algorithm is presented according to the properties of the Bessel function. The products of the Bessel function are expanded to a series, which makes the integral analytic in nature and thereby integrable.

When ωas≤8, the limited series expression can be used as follows:

(11) Jk(as)Jn(bs)=1Γ(n+1)(as2)k(bs2)n×∑q=0∞(−1)q2F1(−q,−k−q;n+1;b2a2)q!Γ(k+q+1)(as2)2q,

where b≤a, Γ(n) is the gamma function and ${}_{2}F_{1}(\alpha ,\beta ,\gamma ,x)$ is the hypergeometric function. When ωas>8, the asymptotic expansion formula of the Bessel function can be used as follows:

(12) $$J_{k}^{Asy}(s)\approx \sqrt{\frac{2}{\pi x}}[\cos(s-\frac{k\pi }{2}-\frac{\pi }{4})\sum_{p=0}^{\infty }\frac{{\left(-1\right)}^{p}(k,2p)}{{\left(2s\right)}^{2p}}-\sin(s-\frac{k\pi }{2}-\frac{\pi }{4})\sum_{p=0}^{\infty }\frac{{\left(-1\right)}^{p}(k,2p+1)}{{\left(2s\right)}^{2p+1}}],$$

According to the numerical analysis, it is accurate enough for *p* = 5 in Equation (12) when k,n≤6.

Substituting Equations (11) and (12) into Equation (3), we can get the analytical and integrable forms of Equation (10) as follows:

(13) $${\left(Y_{kn}^{ij}\right)}^{b}=\sum_{p=0}^{N_{Q}}{\bar{c}}_{p}^{Q}\sum_{l=0}^{N_{kn}}J_{kn}(l)\int_{s_{Q}}^{s_{Q+1}}{\left(\frac{s-s_{oQ}}{\Delta_{Q}}\right)}^{p}{\left(\omega a_{i}s\right)}^{k}{\left(\omega a_{j}s\right)}^{n+2Q}\exp(-js\omega \Delta )ds,$$

or

(14) $${\left(Y_{kn}^{ij}\right)}^{b}=\sum_{p=0}^{N_{Q}}{\bar{c}}_{p}^{Q}\int_{s_{Q}}^{s_{Q+1}}{\left(\frac{s-s_{oQ}}{\Delta_{Q}}\right)}^{p}J_{k}^{Asy}(\omega a_{i}s)J_{n}^{Asy}(\omega a_{j}s)\exp(-js\omega \Delta )ds,$$

In Equation (13), the general form of the integral is $\int_{s_{Q}}^{s_{Q+1}}{\left(\omega a_{i}s\right)}^{t}\exp(-js\omega \Delta )ds$. In Equation (14), the general form of the integral is $\int_{s_{Q}}^{s_{Q+1}}{\left(\omega a_{i}s\right)}^{-t}\exp(-js\omega \Delta )cos[(a_{j}\pm a_{i})\omega s]ds$ or $\int_{s_{Q}}^{s_{Q+1}}{\left(\omega a_{i}s\right)}^{-t}\exp(-js\omega \Delta )\sin[(a_{j}\pm a_{i})\omega s]ds$. These can be calculated using integration by parts for the large value Δ case.

**Figure 5:**
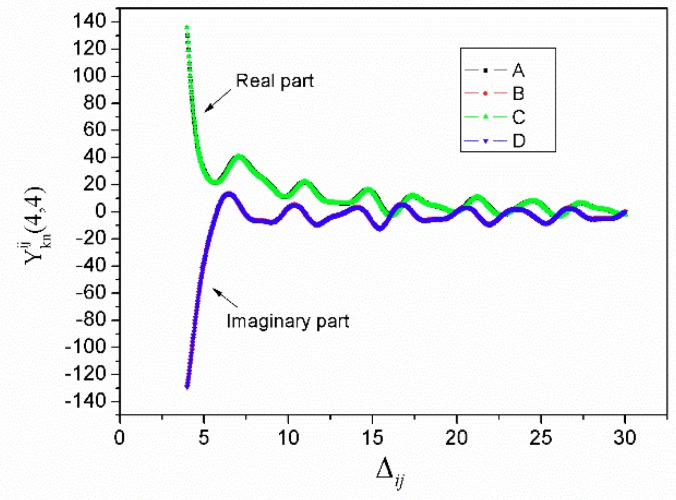
Calculated results of $Y_{kn}^{ij}$ with two algorithms. Curve A and curve B are the results calculated using our algorithm, and curve C and curve D are the results calculated using adaptive quadrature.

Thus, the accurate calculation algorithm of Equation (8) can be obtained using the series expansion of $J_{k}(s\omega a_{i})J_{n}(s\omega a_{j})$ and polynomial interpolation of $\frac{{\bar{H}}^{b}(s)}{\left|s\right|}$. Figure 5 shows the calculated results of $Y_{kn}^{ij}$ with different $\Delta_{ij}$ for different methods. The value of k as well as *n* is 0, as the first series represents the main item. The frequency is close to the center frequency of the transducer. The black line represents the results calculated using our approximation algorithm, and the red and blue lines are the results calculated from adaptive quadrature. The calculation time for our algorithm is one tenth that of the previous algorithm while maintaining the same degree of precision.

## 4. Non-Periodic FEM/BEM Model of the Impedance-Loaded SAW Sensors

After calculating $Y_{kn}^{ij}$, the FEM method was applied to derive the relation between $c_{j}^{u}$ and $B_{kn}^{t}$ [12,13]:

(15) $${\left(c_{j}^{u}\right)}_{i}=Y_{e}B_{kn}^{t}.$$

Equation (3) can be rewritten in the matrix form as

(16) $${\left(\begin{array}{c} c_{j}^{u}\\ c_{j}^{\phi } \end{array}\right)}_{i}=Y\left(\begin{array}{c} B_{kn}^{t}\\ B_{kn}^{\sigma } \end{array}\right)=\left(\begin{array}{cc} Y_{u} & Y_{u\phi }\\ Y_{\phi u} & Y_{\phi } \end{array}\right)\left(\begin{array}{c} B_{kn}^{t}\\ B_{kn}^{\sigma } \end{array}\right),$$

where *Y* is the matrix form of $Y_{kn}^{ij}$, and $Y_{u}$, $Y_{u\phi }$, $Y_{\phi u}$, and $Y_{\phi }$ are matrices related to the mechanical-mechanical, mechanical-electrical, electrical-mechanical and electrical-electrical couplings, respectively.

Substituting Equation (15) into Equation (16), we get

(17) $${\left(\begin{array}{c} 0\\ c_{j}^{\phi } \end{array}\right)}_{i}=\left(\begin{array}{cc} Y_{u}-Y_{e} & Y_{u\phi }\\ Y_{\phi u} & Y_{\phi } \end{array}\right)\left(\begin{array}{c} B_{kn}^{t}\\ B_{kn}^{\sigma } \end{array}\right),$$

**Figure 6:**
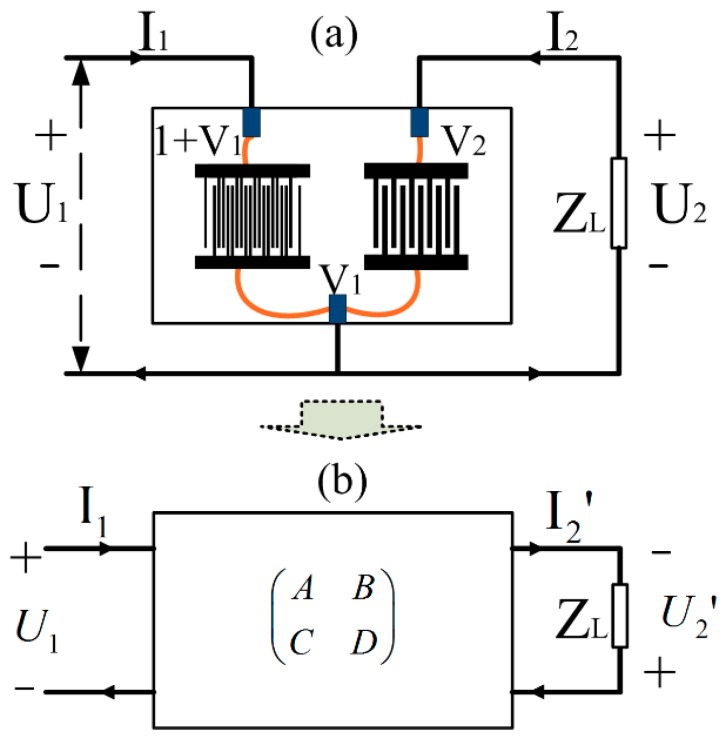
(**a**) Boundary conditions of impedance-loaded SAW sensors; (**b**) ABCD matrix representation of impedance-loaded SAW sensors.

Equation (17) is the system equation for the non-periodic FEM/BEM model. As shown in Figure 6, the electrical boundary conditions of the impedance-loaded SAW sensors will be applied to Equation (17).

The potential of the ground bus bar of the IDTs is noted as *V*_1_ and the potential of the output and input IDTs are noted as *V*_2_ and (1 + *V*_1_), respectively. The omitted time harmonic coefficient is exp(jωt). The condition dictates that the total electric charge must be zero. Substituting the electrical conditions thus gives

(18) $${\left(\begin{array}{c} 0\\ c_{j}^{\phi }\\ 0\\ 0 \end{array}\right)}_{i}=\left(\begin{array}{c} Y_{u}-Y_{e}\\ Y_{\phi u}\\ 0\\ 0 \end{array}\;\begin{array}{c} Y_{u\phi }\\ Y_{\phi }\\ C_{2}\\ C_{4} \end{array}\;\begin{array}{c} 0\\ C_{1}\\ C_{3}\\ C_{5} \end{array}\right)\left(\begin{array}{c} B_{kn}^{t}\\ B_{kn}^{\sigma }\\ V_{1}\\ V_{2} \end{array}\right),$$

where *C*_1_ is a $2\times \sum_{k=1}^{N_{e}}N_{k}^{ch}$ dimension submatrix, *C*_2_ and *C*_4_ are $\sum_{k=1}^{N_{e}}N_{k}^{ch}$ dimensional row vectors, and *C*_3_ and *C*_5_ are two-dimensional row vectors.

For the elements related to the *N*_1_ input IDT in $c_{j}^{\phi }$, the expressions are $c_{j}^{\phi }(N_{1})=\pi$, $C_{1}(N_{1},1)=1$, and $C_{1}(N_{1},2)=0$. For the elements related to the *N*_2_ IDT in $c_{j}^{\phi }$, the expressions are $c_{j}^{\phi }(N_{2})=0$, $C_{1}(N_{2},1)=\pi$, and $C_{1}(N_{2},2)=-\pi$.

*C*_2_ is determined by the charge conservation condition, *C*_3_ = 0, and *C*_4_ and *C*_5_ are determined by $\sum_{k=N_{1}+1}^{N_{e}}a_{k}j\omega \pi B_{k0}^{\sigma }=-\frac{V_{2}-V_{1}}{Z_{L}}$, where *Z_L_* is the impedance of the external loaded sensor.

If *Z_L_* is known, Equation (18) can be solved and Equation (19) is obtained as the final solution.

(19) $$\{\begin{array}{c} U_{1}=1\\ U_{2}=V_{2}-V_{1}\\ I_{1}=\sum_{k=1}^{N_{1}}a_{k}j\omega \pi B_{k,0}^{\sigma }\\ I_{2}=-\sum_{k=N_{1}+1}^{N_{e}}a_{k}j\omega \pi B_{k,0}^{\sigma } \end{array},$$

The outside sensor impedance variation will have a great deal of influence on the electric charge and electric potential distribution. As seen from Equation (18), every time the outside impedance varies, part of the coefficient of the system equations (*C*_4_ and *C*_5_) will change. Thus, the whole system equations must be solved once, which requires a long time for solving the large dimension linear equations. A new strategy was presented in next section.

## 5. Modification of the Non-Periodic Model

In the above method, the SAW sensor was calculated as a unitary device (shown in Figure 6a). In our method, the SAW sensor was treated in a two-port network and the SAW transponder and the loaded impedance sensor part were calculated as a form of the ABCD matrix separately [18] (Figure 6b). This route can isolate the time-consuming large sparse equation-solving task with varying boundary conditions. The ABCD matrix [18] of the SAW chip can be derived using the non-periodic FEM/BEM model under two electrical boundary conditions: Short circuit and open circuit.

When we set the output IDTs as short circuit and open circuit, we can calculate the solutions of Equation (18) for the two situations. Then, we can obtain the expression of the ABCD matrix at all frequency points according to Equation (20). These values in the ABCD matrix are set because it represents the device without considering the external boundary conditions.

(20) $$\left[\begin{array}{c} U_{1}\\ I_{1} \end{array}\right]=\left[\begin{array}{cc} A & B\\ C & D \end{array}\right]\left[\begin{array}{c} U_{2}\\ I_{2} \end{array}\right],$$

When the end of the output IDT is loaded with an impedance *Z_L_*, we can get $I_{2}=U_{2}/Z_{L}$. Substituting $I_{2}=U_{2}/Z_{L}$ into Equation (20), we get

(21) $$\{\begin{array}{c} I_{1}=(C+D/Z_{L})U_{2}\\ U_{1}=(B/Z_{L}+A)U_{2} \end{array},$$

The input impedance of the U_1_ port is $Y=\frac{I_{1}}{U_{1}}=\frac{C+D/Z_{L}}{B/Z_{L}+A}$. Then, the scattering parameters of this special two-port device can be obtained using the conventional formulae.

Using this method, Equation (18) can be solved twice which reduces computation time. This method can also be used in the situation where an impedance matching circuit has an influence on the electrical boundary conditions of the IDT, either at the front or the back ends of the device.

## 6. Experimental Verification

A test device was manufactured to verify the simulated results. The device parameters are as follows: The substrate of the sample is 128° YX-LiNbO_3_. The electrode is aluminium. The periods of the transmitting/receiving IDT and the programmable reflector are both 12.3 µm. The transmitting/receiving IDT has a single finger and the number of finger pairs is 20. The programmable reflector is of the split finger type and the number of finger pairs here is also 20. The metallization ratio of both IDTs is 0.5 and the film thickness is 0.2 µm. The aperture of the IDTs is 421.7 µm. The gap between the IDTs is 3631.8 µm.

**Figure 7:**
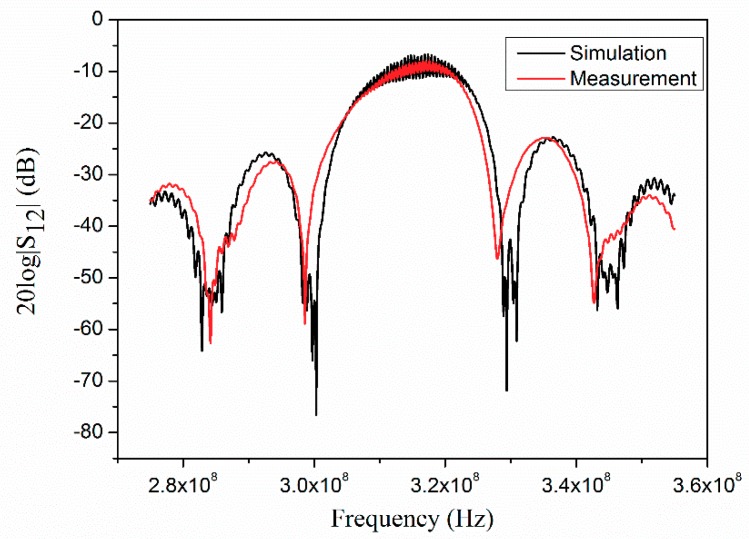
Simulated and measured S_12_ characteristic of the test device.

Using the methods mentioned above, the device performance can be simulated easily. The S_12_ characteristic of the sensor was thus calculated and compared with the measured results in Figure 7. 

It can be seen that the profiles of admittance in the simulation show good similarity with the experimental results. However, the amplitudes at some points are different.

The parameters derived from the above figure are shown in Table 1. The center frequency and bandwidth of the simulation agree well with the measurements. The band ripple of the simulation is larger than the measured values. This is because the test device was measured on a test fixture, where the bond wire effect was not considered in the simulation.

**Figure 8:**
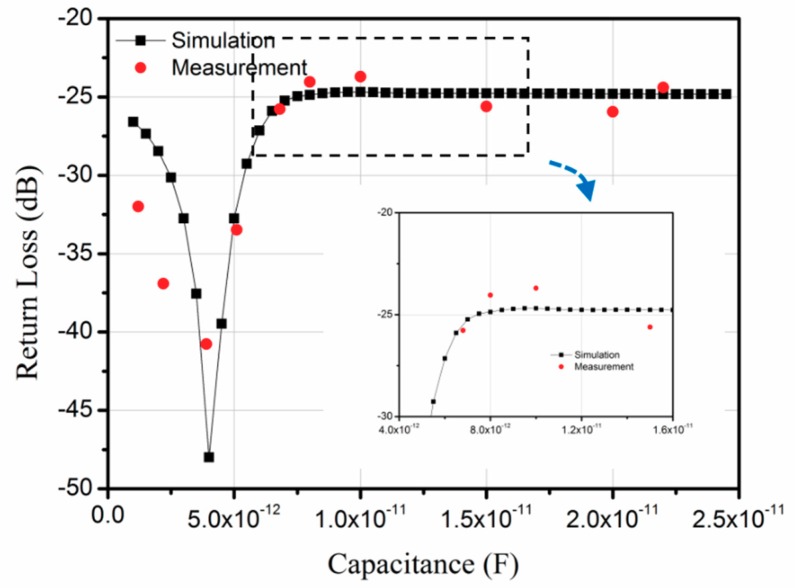
Capacitor dependence of the return loss (*L* = 63 nH, *R* = 1 Ω).

The simulated results, including the loaded matching circuit and capacitive sensor are shown in Figure 8. The return loss is the amplitude of the response pulse in the time domain. The return loss variation curve shows a resonant behavior as presented before [15]. This property was verified by several groups [19,20,21]. The calculated curve corresponds with the trend of the measured points. In the range of 7–12 pF, there is a small ripple in the measured points which can also be seen clearly in the magnified view of the simulated curve. The simulated results are approximately 5 dB lower when the capacitance is 1 pF and 7.5 dB lower for a 2 pF value. This may be because when the capacitance value is small the bond wire effect can easily affect the measured results.

In the last paragraph the bond wire effect was not considered and the profile of the simulated and measured S_12_ curve corresponds perfectly. On the other hand, the simulation result has larger errors for the smaller capacitance value. Both results came to a conclusion that the bond wire effect played an important role in the simulation results, especially for the small impedance value. The next step for our research will be taking this factor into our consideration in the simulation process.

Moreover, the result shows a resonance behavior of the return loss as a function of capacitance of the sensor. The sensor operation could be accepted only on a portion of the capacitance, i.e., in the region of 1–4 pF or 4–7 pF. For the sensing region below 4 pF, the capacitive sensor only can be integrated on the same chip with the SAW sensor to minimize the parasitic capacitance of connecting wires. In addition, it is hard to find a commercial sensor with appropriate capacitance variation. Thus, the need for sensor optimization is urgent in order to shift the monotonous range by trying different sensor configurations. Fast simulation methods will play very important roles in the optimization process.

## 7. Conclusions

The finite transducer analysis method based on the FEM/BEM model, which is one of the most accurate methods for simulating SAW filters, was successfully applied to simulate the impedance-loaded SAW sensors. An accurate and fast algorithm for calculating the bulk wave part of the equation coefficient was presented to reduce the required computing time. Modification to the non-periodic FEM/BEM model was made to adapt to the impedance-loaded sensors. Thus, we only need to calculate the short circuit and open circuit situations of the SAW sensors respectively in order to obtain the sensor response at each value of loaded impedance. The simulated results were compared with the measured results of the test device, which show good accordance.

## Figures and Tables

**Table 1:** Results analysis between the simulation and measurement.

Results Type	Fc (MHz)	Insertion Loss (dB)	3 dB Bandwidth (MHz)	Band Ripple (dB)
Simulation	317.12	8.78	14.2	4.2
Measurement	316.83	9.16	15.1	1.98
Difference	0.09%	−4.1%	−5.9%	52.9%
